# Letter from the Editor in Chief

**DOI:** 10.19102/icrm.2026.17058

**Published:** 2026-05-15

**Authors:** Devi Nair


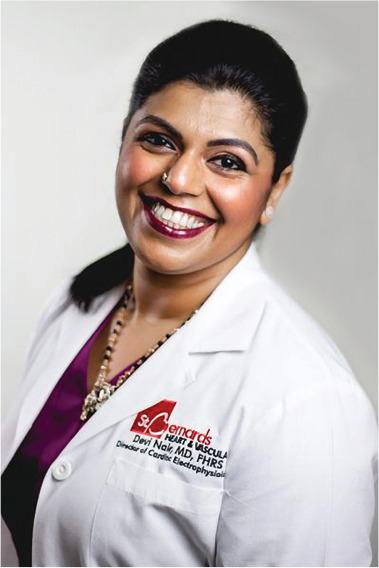


Dear Colleagues,

Welcome to the May 2026 issue of *The Journal of Innovations in Cardiac Rhythm Management*.

May has been an unusually rich month for our community, with three meetings spanning Europe and North America that together traced the contemporary arc of cardiac electrophysiology from bench to bedside to procedural innovation. The nineteenth Annual Scientific Congress of the European Cardiac Arrhythmia Society (ECAS) convened in Munich for a program centered on the science of arrhythmogenesis, ion-channel biology, computational modeling, the mechanistic underpinnings of atrial fibrillation persistence, and the rapidly evolving lesion biology of pulsed field ablation. The twenty-eighth PRAGUE RHYTHM Workshop on Catheter Ablation brought the field back to the laboratory, with live and recorded cases that showcased high-density mapping in complex substrates, conduction system pacing techniques, and the next generation of single-shot and point-by-point platforms. The month closed in Austin, where EP Live offered two intensive days of live and recorded cases curated for the practicing electrophysiologist and fellow-in-training, with a particular emphasis on advances in atrial and ventricular ablation, lead management, and the integration of newer energy sources into established workflows. Three meetings, three temperaments—scientific, technical, and clinical—and a remarkable degree of thematic convergence on the questions that animate our field at this moment.

The manuscripts collected in this issue mirror that convergence. They span original translational science, a contemporary review of defibrillator architecture, a granular examination of conduction system pacing lead durability, novel retrieval techniques for leadless pacemakers, and two case reports that remind us how rare substrates continue to challenge our diagnostic and procedural instincts.

In an original research contribution, Ashour et al.^1^ interrogate the association between systemic inflammation and resting premature beats in 699 older adults drawn from the Midlife in the United States (MIDUS 3) biomarker cohort. Their nonparametric and covariate-adjusted analyses identify elevated interleukin-6 and interleukin-8 levels as significantly associated with both premature atrial and premature ventricular contractions, while interleukin-10, tumor necrosis factor-α, C-reactive protein, and fibrinogen do not track with ectopy. Waist–hip ratio and renal function emerge as independent contributors. The findings extend the growing body of evidence that inflammation is not merely a bystander in atrial and ventricular ectopy but, plausibly, a modifiable substrate, an observation that resonated with much of the basic-science programming at ECAS this month.

Jalil et al.^2^ offer a contemporary review of subcutaneous (S-ICD) and extravascular (EV-ICD) implantable cardioverter-defibrillators, situating these platforms alongside emerging modular cardiac rhythm management systems that pair leadless ventricular pacing with subcutaneous defibrillation through wireless intrabody communication. Their synthesis is candid about what we do and do not yet know: comparative head-to-head data for the EV-ICD against the more mature S-ICD remain limited, and the long-term durability of modular systems will require disciplined registry work and randomized comparisons before broader uptake. For clinicians weighing therapeutic alternatives in patients without indications for transvenous pacing, this review is a clear and current map of a rapidly shifting territory.

Ha et al.^3^ from Monash University report a late distal conductor failure of a Solia S60 stylet-driven lead (Biotronik, Berlin, Germany) deployed for left bundle branch area pacing, identified 2 years after implant in a 79-year-old woman whose presentation followed a syncopal fall and a small-volume subarachnoid hemorrhage. The chest radiograph revealed acute angulation of the lead tip relative to the day of implant, and device interrogation documented a steep impedance rise, loss of R-wave sensing, and exit block at maximal output. The report adds to a small but accumulating series of distal conductor failures in stylet-driven leads intended for the conduction system and is a timely reminder, as conduction system pacing moves rapidly into mainstream practice, that chronic lead behavior in this anatomically constrained territory remains a work in progress. The case fits squarely within the lead-management conversations that ran through PRAGUE RHYTHM and EP Live Austin this month.

Shokr^4^ describes a novel single-tine–based snaring technique for percutaneous retrieval of a Micra™ leadless pacemaker (Medtronic, Minneapolis, MN, USA) complicated by loss of capture 4 days after implant in an 89-year-old man, whose upside-down orientation in the right ventricular outflow tract precluded conventional snaring of the proximal retrieval knob. After multiple attempts with the Aveir™ Retrieval Catheter (Abbott, Chicago, IL, USA) and a Goose Neck snare (Covidien [Medtronic], Dublin, Ireland), engagement of a partially free tine allowed controlled disengagement of the remaining tines, although the device ultimately dislodged into the groin subcutaneous tissue and required a small incision for explantation. The case is a candid account of both the feasibility and the limits of off-label retrieval, and the author’s own caveat, that the tine is durable but that single-tine retrieval should be regarded as a last resort, is the right framing as our leadless populations age and as device exchange becomes a more frequent procedural reality.

Sileshi et al.,^5^ from the University of Kansas, present a remarkable case of out-of-hospital cardiac arrest from ventricular fibrillation in a 26-year-old woman at 34 weeks’ gestation, ultimately attributed to a Wolff–Parkinson–White accessory pathway with a fractionated delta wave arising from a diverticulum of the middle cardiac vein. After three unsuccessful ablation procedures, including attempts with pulsed field energy, cardiac computed tomography angiography disclosed the diverticulum, and irrigated radiofrequency ablation delivered within it eliminated accessory pathway conduction. The authors propose that multiple muscular fibers of the coronary sinus or middle cardiac vein may interface with the ventricle through the diverticulum, generating multiple wavefronts of pre-excitation and producing the unusual delta-wave fractionation that, in retrospect, was the first electrocardiographic clue to an epicardial substrate. The case is a striking example of how the careful re-reading of a 12-lead tracing, paired with cross-sectional imaging, can rescue a procedure that conventional endocardial and pulsed field approaches were unable to complete.

Finally, Masoudkabir et al.^6^ present a case of concomitant short QT syndrome and sick sinus syndrome in a 19-year-old man with recurrent syncope, a corrected QT interval of 318 ms, and a markedly prolonged sinus node recovery time at electrophysiology study. Implantation of an ICD was undertaken in light of the discordant risks his presentation carried: a young patient with overlapping ventricular and bradyarrhythmic vulnerabilities. The report invites a broader reflection on the rare intersections of channelopathy and sinus node dysfunction that occasionally surface in our practices and on the diagnostic and therapeutic decisions they demand.

The May 2026 issue, like the meetings that bracket it, traces the spectrum from biomarker to bedside, from accessory pathway anatomy to the durability of a chronic pacing lead, and from a review of where defibrillation technology is heading to a case report that interrogates where it cannot yet fully reach. The questions raised in these pages, the role of systemic inflammation in atrial and ventricular ectopy, the architecture and longevity of new defibrillator systems, the chronic behavior of conduction system pacing leads, the retrieval calculus for leadless devices, the recognition of epicardial accessory pathways, and the management of overlapping inherited and acquired arrhythmic risk, were precisely the questions that filled the lecture halls of Munich, the demonstration suites of Prague, and the live-case studios of Austin.

I am grateful, as always, to our authors, reviewers, and editorial team for their work, and to you, our readers, for your continued engagement with the journal.

Warm regards,


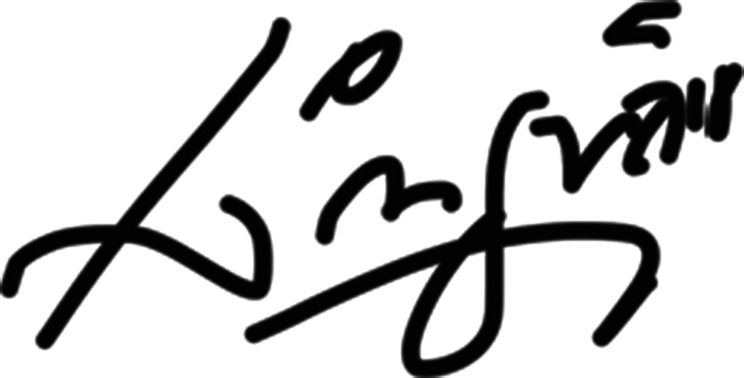


Dr. Devi Nair, md, facc, fhrs

Editor-in-Chief

*The Journal of Innovations in Cardiac Rhythm Management*

Director of the Cardiac Electrophysiology & Research,

St. Bernard’s Heart & Vascular Center, Jonesboro, AR, USA

White River Medical Center, Batesville, AR, USA

President/CEO, Arrhythmia Research Group

Clinical Adjunct Professor, University of Arkansas for Medical Sciences

Governor, Arkansas Chapter of the American College of Cardiology

drdgnair@gmail.com
